# Clinical characteristics of obsessive-compulsive disorder comorbid with obsessive -compulsive personality disorder: subtype implications

**DOI:** 10.3389/fpsyt.2025.1577042

**Published:** 2025-07-14

**Authors:** Ayse Dondu, Levent Sevincok

**Affiliations:** ^1^ Department of Psychiatry, Aydın State Hospital, Aydin, Türkiye; ^2^ Department of Psychiatry, Faculty of Medicine, Adnan Menderes University, Aydin, Türkiye

**Keywords:** obsessive-compulsive disorder, obsessive-compulsive personality disorder, comorbidity, symptom dimensions, early-onset OCD, neurobiology, treatment strategies

## Abstract

**Background:**

Obsessive-compulsive disorder (OCD) is a psychiatric condition characterized by persistent obsessions and compulsions that lead to significant distress and functional impairment. Obsessive-compulsive personality disorder (OCPD) frequently co-occurs in individuals with OCD and has been identified in several studies as the most prevalent personality disorder within this population. However, the clinical relevance of this comorbidity remains uncertain, particularly in terms of whether it reflects a distinct OCD subtype characterized by specific onset patterns and symptom dimensions. The present study aimed to examine whether OCD patients with and without comorbid OCPD differ in terms of age and mode of onset, as well as the content of obsessive-compulsive (OC) symptoms. We hypothesized that the presence of comorbid OCPD may define a clinically meaningful subtype of OCD.

**Methods:**

The sample consisted of 148 individuals diagnosed with OCD, all of whom underwent a comprehensive clinical evaluation. The following clinical variables were recorded: age at onset of OCD, mode of onset (acute vs. insidious), illness course (chronic vs. episodic), total duration of OCD, and other relevant clinical features including symptom severity and treatment history. Diagnostic assessments were conducted according to the criteria of the Diagnostic and Statistical Manual of Mental Disorders, Fifth Edition (DSM-5). In order to evaluate comorbid OCPD, participants were administered the OCPD module of the Structured Clinical Interview for DSM-5 Personality Disorders (SCID- 5-PD), in conjunction with a self-report screener. The severity of current OC symptoms was assessed using the Yale–Brown Obsessive Compulsive Scale (Y-BOCS), while depressive symptoms were measured with the Hamilton Depression Rating Scale (HDRS). Anxiety symptoms were also evaluated using the Hamilton Anxiety Rating Scale (HARS).

**Results:**

Among 148 OCD patients, 58.8% met the diagnostic criteria for OCPD. While no differences were found in demographic variables, those with comorbid OCPD had higher lifetime counts of obsessions and compulsions, greater depression severity, and more severe OC symptoms. They also exhibited an earlier and more insidious onset of OCD and a more chronic illness course. Symmetry and hoarding obsessions, as well as ordering and hoarding compulsions, were more common in this group. Symmetry and hoarding obsessions, as well as ordering and hoarding compulsions, were more prevalent in the comorbid group. Binary logistic regression analysis identified earlier onset (OR = 2.45), insidious onset (OR = 2.68), higher depression severity (OR = 1.15), and presence of ordering compulsions (OR = 2.36) were significantly associated with OCD-OCPD comorbidity.

**Conclusion:**

Comorbid OCD and OCPD may represent a distinct subtype of OCD, characterized by insidious onset, early OC symptom onset, symmetry/ordering, hoarding obsessions and compulsions, and more severe depression. These findings underscore the importance of recognizing heterogeneity within OCD and highlight the need for targeted treatment strategies tailored to distinct clinical subtypes. Future studies should investigate the neurobiological mechanisms and developmental pathways underlying this comorbid presentation, as well as the potential efficacy of cognitive-behavioral interventions specifically designed to address both OC symptoms and maladaptive personality traits.

## Highlights

- Comorbidity may represent a distinct OCD subtype.
- Depression severity is higher in OCD+OCPD group.
- Symmetry and hoarding dimensions are prominent in comorbid group.
- OCD+OCPD patients show earlier and more insidious onset.

## Introduction

OCD is a chronic condition that can, in certain cases, be debilitating, with a global prevalence ranging between 0.5% and 3% among adults (1). The onset of OCD often manifests subtly during childhood and exhibits a protracted course with fluctuating symptoms (2). Extensive research has demonstrated that OCPD ranks among the most prevalent personality disorders in the general population; however, prevalence estimates may vary across studies due to methodological differences, including non-standardized diagnostic tools, cultural factors, and sampling biases (3). According to DSM-IV criteria, the lifetime prevalence of OCPD is estimated to be between 3% and 8% (4). In outpatient settings, OCPD has been identified as the third most frequently diagnosed personality disorder, with a point prevalence of 8.7% based on DSM-IV criteria (5). The connection between OCD and OCPD has been a subject of considerable debate. Studies suggest that 23% to 34% of individuals with OCD also meet DSM-IV criteria for OCPD (6, 7). In a study of 262 participants with OCPD, 20% also met DSM-IV criteria for OCD, as assessed by the SCID (7). Previous research examining the overlap and distinctions between these two disorders has revealed that while rigidity and excessive self-discipline are more characteristic of OCPD, obsessions, particularly those involving contamination and cleaning, are more indicative of OCD (8). Given this high comorbidity rate and the shared features between the disorders, it remains essential to determine whether OCPD defines a clinically meaningful subtype within the OCD population.

Additionally, individuals with both OCD and OCPD are more likely to exhibit compulsions related to cleaning, ordering, repeating, and hoarding, as well as obsessions involving doubt, symmetry, and hoarding (6, 8–11). These individuals also tend to demonstrate lower levels of insight and global functioning (10, 12, 13). Interestingly, no significant differences have been observed between individuals with only OCD and those with comorbid OCPD regarding sex, clinician-rated OCD severity, duration of illness, morbidity risk, disability levels, or family history of tic disorders/Tourette syndrome (10, 11). The literature presents conflicting findings on differences in age of onset and self-reported OCD symptom severity between these groups (10, 11, 13). Some studies suggest that comorbid OCPD may lead to poorer treatment responses in OCD patients, while others found no correlation between OCPD and treatment outcomes (14). Although OCD and OCPD may appear similar, partly due to their diagnostic labels, these similarities might be only superficial. OCD is a chronic and disabling mental disorder, currently classified under the “Obsessive-Compulsive and Related Disorders” category in the DSM-5, and is often characterized by persistent anxiety and distress whereas OCPD is a personality disorder marked by a persistent pattern of excessive attention to detail, rigidity, and perfectionism throughout a person’s life. Historically, it was assumed that OCD would be more prevalent in patients with OCPD than in those with other personality disorders, as an obsessional personality was thought to predispose individuals to OCD. However, since OCPD and OCD are classified separately, some experts argue that these disorders are distinct and unrelated mental illnesses (15). Nevertheless, some researchers speculate that the two conditions share many features, including compulsions, and may be conceptually similar (8, 11). Others even propose that a specific subset of OCD patients also has OCPD (6, 13), or that comorbid OCPD could indicate a more severe form of OCD (10). On the contrary, some scholars suggest that there is no specific link between OCD and OCPD leaving the nature of their relationship uncertain (16)

To our knowledge, the trajectory of OCD in patients with concomitant OCPD has not been thoroughly examined. Certain psychiatric disorders, such as schizophrenia, are believed to be influenced by the subtle or sudden onset of symptoms. OCD can present with either an acute or insidious onset, with most patients reporting a gradual onset. However, some studies suggest that a significant proportion of patients experience a sudden onset, with 50-70% reporting that this onset was preceded by stressful life events (17). Sudden onset of OCD symptoms may be linked to underlying organic causes, such as infections triggering OCD-like behaviors.

In light of the limited understanding of the relationship between OCPD and the onset of OCD symptoms (acute or insidious), the objective of this study was to ascertain whether OCD patients with and without OCPD differ in terms of the type of OCD symptoms they present with, their age at onset, the course of their illness, and the content of their OCD symptoms. It was hypothesized that the co-occurrence of OCPD in individuals with OCD may reflect a clinically meaningful subtype, particularly if specific patterns related to onset characteristics or symptom profiles distinguish this group from non-comorbid cases.Methods

### Ethical approval and participants

Ethical approval for this study was obtained from the Non-interventional Ethics Committee (#2016/904) of the Faculty of Medicine at Aydin Adnan Menderes University. Informed voluntary consent forms were obtained from all participants before the study. The study was performed in accordance with the Helsinki Declaration.

A total of 199 patients diagnosed with OCD according to DSM-5 criteria were recruited from the outpatient psychiatry clinic of Aydın State Hospital, with diagnoses established through initial clinical interviews conducted by trained psychiatrists. Patients with OCD who were between the ages of 18 and 65 and did not have schizophrenia, bipolar disorder, intellectual disability, substance abuse problems, or a history of any neurological diseases other than tic disorders were eligible for inclusion. Comorbid OCPD was assessed using the OCPD module of the Structured Clinical Interview for DSM-5 Personality Disorders (SCID-5-PD), alongside a validated self-report screener. 51 of the patients who were initially evaluated were excluded from the final sample because the diagnostic interview and assessment tests could not be completed, making it impossible to determine the presence of OCPD. Prior to exclusion, these individuals were compared with the included sample in terms of age, education level, and symptom severity, and similar clinical or demographic variables, and no statistically significant differences were observed. Thus, their exclusion did not compromise the representativeness or the internal validity of the final study sample. The clinical variables of age at OCD onset, mode of onset (acute or insidious), and illness course (chronic or episodic) were documented for 148 OCD participants. These data were based on retrospective self-reports, which may carry a risk of recall bias.

Participants were asked to report the mode of onset as acute (if OC symptoms appeared within one month) or insidious (if there was a gradual transition from premorbid personality to overt OCD over more than three months), as defined in previous studies. The age at onset and the duration of OCD were determined from the age that the patient remembered as the beginning of the OC symptoms. Participants who retrospectively reported clinically significant OCD for at least two consecutive years before baseline were classified as having chronic OCD, consistent with previous studies (18). The patients were recorded as having episodic OCD if they had a symptom-free interval of at least one month in a year with or without treatment. OC symptoms severity was measured using the Yale–Brown Obsessive Compulsive Scale (Y-BOCS) for OCD, the Hamilton Depression Rating Scale (HDRS) for depressive symptoms, and the Hamilton Anxiety Rating Scale (HARS) for anxiety symptoms.

### Measures

The diagnosis of OCPD was assessed using the SCID-5-PD. The Turkish version (SCID-5-PD-CV-TR) was developed under the coordination of Bayad et al. and validated across two university hospitals with 102 participants (19). Inter-rater reliability was found to be substantial, with Cohen’s Kappa coefficients ranging from 0.64 to 0.90 across all personality disorder modules, and a Kappa of 0.81 specifically for OCPD. The instrument also demonstrated robust convergent and divergent validity, confirming its utility in Turkish clinical settings.

The severity of OC symptoms was evaluated using the Y-BOCS. The Turkish adaptation of Y-BOCS has shown strong internal consistency (Cronbach’s α = 0.88) and excellent test-retest reliability (r = 0.98) (20). Depressive symptoms were assessed with the HDRS, which also demonstrates satisfactory psychometric properties in Turkish samples, with an internal consistency of α = 0.75 and inter-rater reliability of r = 0.90 (21). Anxiety symptoms were evaluated using the HARS, which has been validated in Turkish populations, demonstrating strong internal consistency (Cronbach’s α = 0.79) and high test-retest reliability (r = 0.91) (22).

### Data analysis

Statistical analyses were conducted using SPSS software (Version 21.0; SPSS Inc., Chicago, Illinois, USA). The Kolmogorov–Smirnov test was conducted to assess the normality of continuous variables, including age, Y-BOCS total and subscale scores, HDRS, and HARS scores. As all variables were found to be normally distributed (p > 0.05), independent samples t-tests were used for continuous variables. Categorical variables were analyzed using the Chi-square test.

To identify characteristics associated with the comorbidity of OCD and OCPD in 148 patients diagnosed with OCD, a binary logistic regression model was employed. The results were reported as odds ratios (ORs) with 95% confidence intervals (CI). The Hosmer-Lemeshow test was used to assess the goodness-of-fit of the binary logistic regression model. All statistical tests were two-tailed, and a p-value of <0.05 was considered statistically significant.

## Results

### Sociodemographic and clinical characteristics

The sample consisted of 148 individuals diagnosed with OCD, of whom 87 (58.8%) met criteria for comorbid OCPD and 61 (41.2%) did not. There were no significant differences between the OCD+OCPD and OCD-only groups in terms of sex distribution (χ² = 0.018, p = 0.894) or marital status (χ² = 0.926, p = 0.819). Similarly, no differences were found in age (M = 31.33 ± 10.85 vs. 30.65 ± 9.55; t = 0.390, p = 0.697) or years of education (M = 10.80 ± 3.91 vs. 10.88 ± 3.61; t = -0.127, p = 0.899) (**Table 1**).

**Table 1:** Sociodemographic characteristics of OCD patients with and without OCPD.

Variable	OCD+OCPD (n=87)	OCD (n=61)	χ²/t	p
Sex (Female/Male)	59/28	42/19	0.018	0.894
Marital Status (Married/Single/Divorced/Widowed)	43/40/2/1	29/30/2/0	0.926	0.819
Age (years; Mean ± SD)	31.33 ± 10.85	30.65 ± 9.55	0.390	0.697
Education (years; Mean ± SD)	10.80 ± 3.91	10.88 ± 3.61	-0.127	0.899

Group comparisons were performed using chi-square tests for categorical variables and independent samples t-tests for continuous variables.

However, participants with OCD+OCPD were significantly more likely to exhibit an insidious onset pattern (χ² = 10.477, *p* = 0.001) and to report a chronic illness course (χ² = 4.196, *p* = 0.041). The presence of a triggering life event (χ² = 1.827, *p* = 0.401) and history of tics (χ² = 0.848, *p* = 0.357) did not significantly differ between groups. The groups did not differ significantly in terms of having a history of depression (χ² = 3.168, *p* = 0.075) (**Table 2**).

**Table 2:** Clinical and psychometric characteristics of OCD patients with and without OCPD.

Variable	OCD+OCPD (n=87)	OCD (n=61)	χ²/t	p
History of Depression (No/Yes)	26/38	8/27	3.168	0.075
Onset Type (Sudden/Insidious)	6/57	18/35	10.477	0.001
Triggering Life Event (No/Yes)	25/39	16/37	1.827	0.401
Course (Chronic/Episodic)	48/17	29/23	4.196	0.041
History of Tics (No/Yes)	48/14	43/8	0.848	0.357
HDRS Score (Mean ± SD)	19.96 ± 10.55	14.74 ± 10.11	2.843	0.005
HARS Score (Mean ± SD)	15.11 ± 8.08	11.76 ± 8.12	2.401	0.018
OCD Duration (years)	11.61 ± 9.20	8.13 ± 6.94	2.475	0.015
Untreated Duration (years)	6.05 ± 7.49	4.62 ± 6.08	1.218	0.225
Y-BOCS Total	25.80 ± 6.51	21.77 ± 8.34	3.148	0.002
Y-BOCS Obsession	12.99 ± 3.56	11.30 ± 4.46	2.447	0.016
Y-BOCS Compulsion	12.81 ± 3.35	10.72 ± 4.44	3.116	0.002
Past Obsession Count	3.12 ± 1.28	2.61 ± 1.26	2.400	0.018
Current Obsession Count	2.87 ± 1.27	2.20 ± 1.25	3.207	0.002
Past Compulsion Count	2.74 ± 1.28	2.25 ± 1.12	2.452	0.015
Current Compulsion Count	2.56 ± 1.31	1.98 ± 1.30	2.661	0.009

HARS, Hamilton Anxiety Rating Scale; HDRS, Hamilton Depression Rating Scale; Y-BOCS, Yale–Brown Obsessive Compulsive Scale. Statistical tests include chi-square for categorical variables and independent samples t-tests for continuous measures.

When compared using continuous clinical measures, the OCD+OCPD group exhibited significantly higher scores on the HARS (*M* = 19.96, *SD* = 10.55) than the OCD-only group (*M* = 14.74, *SD* = 10.11), *t* = 2.843, *p* = 0.005. Similarly, the HDRS scores were elevated in the comorbid group (*M* = 15.11 vs. 11.76), *t* = 2.401, *p* = 0.018 (**Table 2**). The OCD+OCPD group had a significantly earlier age of OCD onset (M = 19.31 vs. 22.63; t = -2.178, p = 0.031) and a longer illness duration (M = 11.61 vs. 8.13 years; t = 2.475, p = 0.015).The total Y-BOCS scores (*M* = 25.80 vs. 21.77), *t* = 3.148, *p* = 0.002, as well as both obsession (*t* = 2.447, *p* = 0.016) and compulsion subscale scores (*t* = 3.116, *p* = 0.002), were significantly higher in the OCD+OCPD group.

Additionally, the comorbid group showed a significantly greater number of both past (t = 2.400, p = 0.018) and current (t = 3.207, p = 0.002) obsessions, as well as more current (t = 2.661, p = 0.009) and past (t = 2.452, p = 0.015) compulsions (**Table 2**).

### Obsession and compulsion subtypes

Detailed comparisons of obsession and compulsion subtypes between the OCD+OCPD and OCD-only groups are presented in **Table 3**. The analysis revealed that symmetry-related obsessions were significantly more prevalent in the OCD+OCPD group both in the past (58.6% vs. 34.4%, χ² = 8.84, *p* = 0.003) and currently (57.5% vs. 26.2%, χ² = 14.17, *p* < 0.001). Similarly, hoarding obsessions were also more common among individuals with comorbid OCPD in both past (31.4% vs. 11.5%, χ² = 7.97, *p* = 0.005) and current reports (28.7% vs. 8.3%, χ² = 9.10, *p* = 0.003).Among compulsions, ordering behaviors were significantly more frequent in the OCD+OCPD group (58.6% vs. 27.9%, χ² = 13.65, *p* < 0.001), as were hoarding-related compulsions (28.7% vs. 8.2%, χ² = 9.36, *p* = 0.002). Other compulsive behaviors also showed significant group differences (32.2% vs. 16.4%, χ² = 5.39, *p* = 0.020). No statistically significant differences were found between groups in terms of aggressive, religious, sexual, or somatic obsessions, nor for common compulsions such as checking, cleaning, repeating, or counting (**Table 3**).

**Table 3:** Distribution of obsession and compulsion subtypes in OCD patients with and without OCPD.

Variable	OCD+OCPD (n=87)	OCD (n=61)	χ²	p
Obsessions – Past				
Aggressive	29 (33.7%)	16 (26.2%)	0.94	0.332
Contamination	64 (74.4%)	48 (78.7%)	0.36	0.549
Symmetry	51 (58.6%)	21 (34.4%)	8.84	0.003
Hoarding	27 (31.4%)	7 (11.5%)	7.97	0.005
Sexual	10 (11.6%)	5 (8.2%)	0.46	0.498
Religious	18 (20.9%)	15 (24.6%)	0.28	0.600
Somatic	9 (10.5%)	11 (18.0%)	1.74	0.187
Other	61 (70.9%)	37 (60.7%)	1.70	0.193
Obsessions –Current				
Aggressive	27 (31.0%)	11 (18.0%)	3.18	0.075
Contamination	60 (69.0%)	42 (68.9%)	0.00	0.988
Symmetry	50 (57.5%)	16 (26.2%)	14.17	<0.001
Hoarding	25 (28.7%)	5 (8.3%)	9.10	0.003
Sexual	9 (10.3%)	4 (6.6%)	0.64	0.423
Religious	17 (19.5%)	12 (19.7%)	0.00	0.984
Somatic	8 (9.2%)	9 (14.8%)	1.09	0.297
Other	55 (63.2%)	34 (55.7%)	0.84	0.360

Chi-square tests were used to compare categorical variables between groups.

Results from the Binary logistic regression analysis indicate that earlier age at onset (B = -0.121, Exp(B) 95% CI = 0.886; 0.823-0.954; p=0.001), insidious onset (B =-1.473, Exp(B) 95% CI =0.229; 0.061-0.862; p=0.029), HDRS scores (B = 0.098, Exp(B) 95% CI = 1.103; 1.028-1.182; p=0.006), and ordering compulsions (B = -1.952, Exp(B) 95% CI = 0.142; 0.036-0.561; p=0.005) were significantly associated with OCD and OCPD comorbidity (**Table 4**).

**Table 4:** The logistic regression analysis among other variables in the comorbidity of OCD and OCPD.

Variable	B (Coefficient)	Exp (B)	95% CI (Confidence Interval)	p-value
Early Age at Onset	-0.121	0.886	0.823 - 0.954	0.001
Insidious Onset	-1.473	0.229	0.061 - 0.862	0.029
HDRS Scores	0.098	1.103	1.028 - 1.182	0.006
Ordering Compulsions	-1.952	0.142	0.036 - 0.561	0.005

Logistic regression analysis was used to examine factors associated with comorbid obsessive-compulsive personality disorder (OCPD) in individuals diagnosed with obsessive-compulsive disorder (OCD). *B*, unstandardized regression coefficient; Exp(B), odds ratio; CI, confidence interval. *p* values <.05 are considered statistically significant.

## Discussion

In this study, we specifically investigated whether OCD patients with and without comorbid OCPD differ in terms of the mode of onset, age at onset, and content of OC symptoms. We hypothesized that our findings would suggest that comorbidity with OCPD could represent a distinct subtype of OCD. Of the 148 participants diagnosed with OCD, 58.5% (n = 86) also fulfilled the DSM-5 criteria for OCPD. This prevalence rate is significantly higher than the 22.9% to 34.7% range reported in previous studies (6, 13). Variations in OCPD prevalence rates may be attributed to differences in diagnostic tools and thresholds used across studies. The distinction between OCD and OCPD is often considered to be that OCD symptoms are predominantly ego-dystonic, while OCPD symptoms are primarily ego- syntonic.

The findings of this study suggest that individuals with comorbid OCD and OCPD experience a more insidious and earlier onset of OCD, a more protracted illness course, and elevated levels of OCD severity and current depression. They also exhibit a higher prevalence of symmetry and hoarding obsessions, as well as ordering and hoarding compulsions. However, when controlling for factors such as onset type, age at onset, OC symptoms, and current depression severity, chronicity of OCD was not a predictor of comorbidity.

Instead, differences between OCD patients with and without OCPD were predominantly associated with the insidious and earlier onset of OC symptoms and ordering compulsions. These findings align with previous studies suggesting that OCD patients with comorbid OCPD tend to have an earlier onset of OC symptoms (6, 9, 13). The early onset of OC symptoms in comorbid cases raises the question of shared neurobiological mechanisms between OCD and OCPD. Structural and functional neuroimaging studies have shown that both disorders involve abnormalities in the frontostriatal circuitry, suggesting a possible neurodevelopmental link between them. Further research is needed to determine whether these neural correlates could serve as potential biomarkers for this comorbid subtype.

The early onset and insidious progression of OCD symptoms in individuals with comorbid OCPD suggest a potential neurodevelopmental component to this subtype. This trajectory raises intriguing parallels with neurodevelopmental disorders such as autism spectrum disorder (ASD) and attention-deficit/hyperactivity disorder (ADHD), which are also characterized by early onset, persistent symptoms, and functional impairments linked to frontostriatal circuit dysfunction (23, 24). Moreover, these disorders share genetic vulnerabilities, including polymorphisms in serotonergic (e*g.*, SLC6A4*)* and dopaminergic (e.g., COMT*)* genes, which have also been implicated in OCD and OCPD (25, 26). Such overlap may point to shared neurobiological pathways and developmental mechanisms underlying these conditions. Exploring these connections further could enrich our understanding of OCD-OCPD comorbidity and support the development of transdiagnostic models that bridge personality pathology and neurodevelopmental risk (27).

Emerging evidence suggests that genetic and epigenetic factors may contribute to the co-occurrence of OCD and OCPD. Recent findings suggest that OCPD may share neurobiological and genetic underpinnings with OCD. Structural and functional abnormalities in frontostriatal circuitry—particularly in the dorsolateral prefrontal cortex, orbitofrontal cortex, and anterior cingulate cortex—have been observed in individuals with OCPD, suggesting overlapping neural pathways with OCD (28). The structural and functional abnormalities in prefrontal and frontostriatal regions reported in individuals with OCPD may reflect early-emerging cognitive rigidity and perfectionism traits that contribute to the insidious onset and chronic course observed in comorbid OCD-OCPD cases. Given that these neural alterations are also implicated in neurodevelopmental trajectories, it is plausible that the presence of OCPD traits from adolescence or early adulthood may predispose individuals to an earlier and more persistent manifestation of OCD. This interpretation is consistent with our findings, where comorbid cases exhibited significantly earlier onset and more insidious progression of OC symptoms, suggesting a potential neurodevelopmental underpinning aligned with the frontostriatal dysfunction(28).

In addition, polymorphisms in genes regulating serotonergic function, such as the 5-HTTLPR variant in the *SLC6A4* gene, have been associated with obsessive-compulsive personality traits in community samples (29) reinforcing the role of serotonergic vulnerability in personality pathology. Furthermore, OCPD may not be a unitary construct; dimensional models propose two major components—perfectionism/control and interpersonal rigidity—each potentially linked to distinct genetic and neurodevelopmental profiles (30).Taken together, these findings support the hypothesis that OCPD and OCD may arise from partially shared neurobiological and genetic mechanisms, potentially contributing to their frequent comorbidity. Beyond genetic influences, epigenetic mechanisms such as DNA methylation may also play a critical role in mediating the relationship between environmental stressors and gene expression. These processes could explain how early-life adversity or chronic stress modulates the phenotypic expression of obsessive-compulsive traits in vulnerable individuals. Specifically, methylation patterns within the promoter region of SLC6A4 have been shown to affect stress reactivity and serotonin transporter expression, highlighting the importance of gene–environment interactions in the pathophysiology of OCD-related conditions (31, 32). Incorporating these biological frameworks into the conceptualization of OCD-OCPD comorbidity may offer deeper insight into their shared etiology and developmental trajectory.

Data on the onset type of OCD symptoms in patients with comorbid OCPD remain limited. Garyfallos et al. found no difference in the gradual versus abrupt onset of OCD between patients with and without OCPD (13). In contrast, a study involving adult OCD patients reported that 81% experienced a gradual onset of symptoms. Recent research with children found that 24% experienced a rapid onset, while 50% had a gradual onset, with the latter group tending to develop OCD at a younger age (33). Millet et al. (2004) identified a rapid onset and higher prevalence of pre-existing depression in patients with late-onset OCD (34). Additionally, some studies suggest that females with OCD are more likely than males to report stressful experiences and an acute onset (35). Research has also found that individuals with washing and checking compulsions may experience a more insidious onset of OCD symptoms (17). Our study’s finding that insidious onset is more frequent in comorbid OCD-OCPD cases suggests that underlying temperamental factors, such as higher harm avoidance and perfectionism, could contribute to this progression. Recent findings suggest that temperamental traits such as harm avoidance and perfectionism, which are frequently observed in individuals with OCD and OCPD, may also be common across neurodevelopmental disorders ASD. These traits, especially cognitive rigidity and repetitive behaviors, represent shared dimensions that transcend traditional diagnostic boundaries. For instance, Gadelkarim et al. (2019) found significant overlaps between OC personality traits and autistic features among OCD outpatients, supporting the hypothesis of a shared neurodevelopmental substrate (36). Similarly, Bejerot (2007) proposed an “autistic dimension” within OCD, suggesting a potential subtype characterized by inflexibility and resistance to change (37). Furthermore, Dingemans et al. (2022), using network analysis, emphasized the interconnectivity of obsessive-compulsive spectrum disorders, including traits seen in both OCPD and ASD (38). In line with this, Bejerot and Mörtberg (2009) demonstrated that autistic traits could influence social outcomes in individuals with OCD and social anxiety, reinforcing the transdiagnostic relevance of such temperamental vulnerabilities (39).Taken together, these studies highlight the importance of examining shared cognitive and personality dimensions in the etiology and development of comorbid OCD and OCPD, potentially reflecting a broader neurodevelopmental continuum.

Future studies should explore whether these traits mediate the relationship between early-onset OCD and the later emergence of OCPD features. Most research indicates that men experience a longer, more chronic course and an earlier, more subtle onset of OCD compared to women (35, 40, 41). Prior studies suggest that individuals with impulse control issues who suffer from OCD tend to develop the condition at a younger age, with symptoms manifesting more subtly (40, 41). Pediatric Autoimmune Neuropsychiatric Disorders Associated with Streptococcal Infection

(PANDAS) represent a subset of pediatric OCD, characterized by early onset and an episodic course following streptococcal infection (42). Contrary to previous research that did not find a link between comorbid OCPD and increased overall OCD severity, our findings suggest that the presence of both OCD and OCPD is associated with higher levels of self-reported OCD severity, in line with Lochner et al. (10). Additionally, several studies have indicated that individuals with OCPD tend to exhibit more severe compulsions (6, 13). Given the observed increase in OCD severity in comorbid cases, clinicians should consider targeted interventions that address both compulsive behavios and maladaptive personality traits. Cognitive-behavioral therapy (CBT) protocols that incorporate schema-focused interventions may offer particular benefit for individuals with comorbid OCD and OCPD. As Pinto et al. (2022) emphasize, schema-based approaches are well-suited to address maladaptive cognitive patterns such as perfectionism and inflexibility, which are commonly seen in OCPD and may limit response to standard CBT (43). Furthermore, Finch et al. (2021) support the application of Good Psychiatric Management (GPM) and schema-informed therapy as effective frameworks for treating OCPD, particularly in cases involving persistent interpersonal difficulties and chronic functional impairment (44).These integrative approaches may therefore be especially relevant in the treatment of comorbid OCD-OCPD presentations. Specifically, schema-focused techniques such as limited reparenting, mode work, and cognitive restructuring can help address the early maladaptive schemas underlying both personality traits and OC symptoms, thereby enhancing CBT outcomes (43, 44).

Our study found that lifetime obsessions with symmetry and hoarding, as well as compulsions related to ordering and hoarding, were significantly more common among patients with comorbid OCD and OCPD. Notably, ordering compulsions emerged as a predictor of comorbidity. These findings are consistent with prior research indicating that the presence of OCPD is associated with a higher frequency of certain obsessions and compulsions, including those related to contamination, religion, symmetry, ordering, and hoarding (6, 10, 13). Previous studies have highlighted that the presence of comorbid OCPD in OCD patients is associated with a distinct symptom profile and increased clinical severity. Coles et al. found that individuals with both OCD and OCPD were more likely to report ordering, symmetry, and hoarding symptoms, suggesting that these dimensions may serve as markers of personality comorbidity within OCD. Similarly, Lochner et al. reported that OCD patients with comorbid OCPD exhibited significantly higher severity scores on the Y-BOCS, greater depressive and anxiety symptoms, and lower overall functioning compared to those without OCPD. Their findings support the notion that OCPD comorbidity may indicate a more severe and impairing OCD subtype, particularly characterized by compulsions involving order and symmetry (6, 10). The identification of these dimensions highlights the relevance of differentiating OCD subtypes, especially in the context of tailoring interventions for patients with pronounced ordering behaviors and rigid cognitive patterns

A significant finding of this study is that comorbid OCD patients had higher current depression scores compared to those without OCPD. The severity of current depression was significantly associated with the comorbidity of both disorders, even when controlling for other variables. Previous research has similarly reported greater depression in OCD patients with comorbid OCPD (9). It is possible that perfectionism and inflexible standards—core features of OCPD—contribute to increased vulnerability to depressive affect. Conversely, persistent depressive symptoms may exacerbate self-critical cognitive styles, reinforcing maladaptive personality traits. Future research should explore the bidirectional nature of this relationship and assess whether emotion regulation deficits mediate this comorbidity

Earlier reports suggest that OCD patients are more frequently associated with dependent or avoidant personality disorders than with OCPD. It has been noted that OCD does not fit the description of an OCPD diathesis, and there are hypothesized links between OCPD and various anxiety and mood disorders. Prior research presents divergent conceptualizations of the relationship between OCD and OCPD. Some studies have highlighted the temporal stability and diagnostic specificity of OCPD traits, supporting the notion that comorbid OCPD may constitute a clinically meaningful OCD subtype (15). In contrast, other frameworks argue that shared characteristics such as perfectionism, cognitive rigidity, and need for control may reflect dimensional personality traits that overlap with OCD without necessarily indicating distinct comorbidity (16). These contrasting perspectives underscore the complexity of the OCD-OCPD relationship and point to the potential utility of integrating categorical and dimensional models to better capture this heterogeneity in future research.

Overall, our findings suggest that the comorbidity of OCD and OCPD may identify a distinct subtype of OCD, characterized by insidious onset, early OC symptom onset, ordering compulsions, and more severe depression. Further longitudinal studies are needed to establish the developmental trajectory of comorbid OCD-OCPD and determine whether early interventions targeting obsessive-compulsive traits could mitigate the emergence of personality pathology. These results provide further support for the notion that comorbid OCPD may represent a specific clinical subtype of OCD, with potential implications for etiology, course, and treatment responsiveness

Several conceptual frameworks, including “spectrum” and “predisposition” models, have been proposed to explain the co-occurrence of personality disorders (28, 45).We propose that the phenomenological distinctions between OCD patients with and without OCPD may indicate a different etiology. The presence of one disorder may increase the likelihood of developing a second disorder (46–48).

Zhang et al. (2024) reported that individuals with comorbid OCD and OCPD not only exhibited higher severity of obsessions but also showed significant structural brain differences, particularly in regions such as the left superior parietal lobule and precuneus. These areas are implicated in cognitive flexibility, internal attention regulation, and visuospatial organization, and are thought to play a central role in symmetry- and ordering-related behaviors. These neuroanatomical findings align with the clinical profile observed in our study, which was characterized by early onset, insidious progression, and prominent symmetry/ordering compulsions. Taken together, these results suggest that the OCD+OCPD comorbidity may represent a distinct subtype not only phenotypically but also at the neurodevelopmental level. This highlights the need for further research into the neurobiological underpinnings of early-onset obsessive-compulsive symptoms within this comorbid presentation (47).

The findings of this study align with and extend prior research indicating that OCD patients with comorbid OCPD present with a more severe clinical profile compared to those without the personality disorder. The higher Y-BOCS total and compulsion scores observed in the OCD+OCPD group mirror recent reports, including Zhang et al. (2024) who found increased obsessive beliefs, anxiety, and depression levels among comorbid individuals, along with alterations in cortical complexity in regions such as the left superior parietal lobule and precuneus (49). These neural findings may reflect the rigid cognitive style and heightened need for control characteristic of OCPD, which can amplify obsessive-compulsive symptom expression. Consistent with the multidimensional model of OCD proposed by Mataix-Cols et al. (2005), our study also highlights the heterogeneity of symptom presentation, with specific dimensions like symmetry/ordering and hoarding more prevalent in the comorbid group. Such dimensions have been linked not only to earlier age of onset but also to poorer treatment response and increased comorbidity, suggesting the potential utility dimension-specific clinical strategies (50).

Furthermore, Alemany-Navarro et al. provided compelling genetic evidence supporting the specificity of these dimensions. Their pilot GWAS identified genes differentially associated with hoarding, aggressive/checking, and symmetry/order dimensions—findings that may partially explain the clinical variation observed across OCD presentations. Our results are consistent with this line of evidence, as patients with comorbid OCPD demonstrated a symptom pattern marked by greater dimensional diversity and persistence (51).

Interestingly, the impact of genetic risk was further addressed in a separate study by the same group, showing that polygenic risk scores (PRS) for OCD severity were predictive of symptom levels, though not of pharmacological response per se. This aligns with our findings, where symptom severity in the OCD+OCPD group was significantly higher, suggesting that comorbid personality pathology may interact with genetic susceptibility to exacerbate the disorder (52).

Collectively, these studies support the need to move beyond categorical diagnosis toward a more nuanced, dimensional and transdiagnostic framework in both research and clinical practice. The integration of personality traits, symptom dimensions, and neurobiological data—along with consideration of early developmental and genetic factors—could pave the way for more targeted interventions and refined nosology. Our findings further corroborate the importance of symptom dimension differentiation in understanding OCD, especially in the context of comorbid personality pathology such as OCPD. Alemany-Navarro et al. provided robust evidence for distinct genomic correlates of OCD dimensions, identifying genes like SETD3 and CPE as significantly associated with hoarding and aggressive symptoms, respectively. These results reinforce the notion that each OCD dimension may follow a partially unique biological trajectory (53). Critically, the symmetry/order and hoarding dimensions—shown in our study to be more prevalent in the OCD+OCPD group—were also found to have distinct functional pathway signatures, including those related to lipid metabolism, neurodevelopment, and ion transport. The same study highlighted that these dimensions tend to cluster with early-onset cases and have been associated with poorer pharmacological response, emphasizing the clinical utility of recognizing dimensional overlaps in OCD+OCPD presentations.

Although the GWAS by Strom et al. (2024) did not explicitly examine OCPD, the identification of polygenic influences on obsessive-compulsive traits raises the possibility that overlapping genetic mechanisms may also contribute to rigid personality features observed in comorbid cases (54).

Moreover, recent genetic evidence from the same cohort suggests the involvement of serotonin transporter polymorphisms (SERTPR), COMT variants, and estrogen receptor haplotypes in influencing dimensional severity, particularly among patients with symmetry/order, religious, and hoarding symptoms (53). These patterns may explain the neurodevelopmental and treatment-resistant characteristics we observed in the OCD+OCPD subgroup.

Thus, converging genetic, clinical, and symptom-based findings point toward a neurobiologically distinct endophenotype within OCD+OCPD individuals, warranting early identification and potentially tailored intervention strategies.

Emerging evidence highlights the importance of early identification and intervention in disorders that share neurobiological vulnerabilities, including OCD and OCPD. Cioni et al. (2016) emphasized the role of early enriched environments in promoting neural plasticity and improving outcomes in neurodevelopmental disorders (55). In line with this, Hegemann et al. (2024) demonstrated significant genetic and phenotypic heterogeneity in early neurodevelopmental traits, particularly those related to executive functioning and cognitive flexibility (56). Emotion regulation has also been identified as a transdiagnostic factor across conditions such as OCD, OCPD, ASD, and ADHD, with Cai et al. (2024) providing a comprehensive overview of current intervention strategies in this domain (57). Furthermore, Riccio and Gomes (2013) reviewed effective interventions for executive function deficits in children and adolescents, including metacognitive and computerized approaches (58). Similarly, MacPherson et al. (2013) showed that dialectical behavior therapy (DBT), adapted for adolescents, can improve emotion regulation and executive skills (59). Together, these findings suggest that early interventions targeting core neurocognitive and affective processes may hold promise for individuals with comorbid OCD and OCPD and should be further explored in future research.

## Conclusions

Future research should prioritize longitudinal studies that track the developmental trajectory of OCD and OCPD from childhood to adulthood. Such designs would allow researchers to determine whether early neurocognitive or behavioral markers can predict the emergence of comorbid OCPD features in individuals with OCD. Neuroimaging studies may be particularly useful in examining whether early alterations in frontostriatal connectivity are associated with the later development of personality rigidity and perfectionism. Additionally, genetic and epigenetic studies could identify specific biomarkers, that differentiate comorbid cases from non-comorbid OCD. These approaches would contribute to a more precise understanding of the etiological mechanisms underlying this complex comorbidity and inform personalized early intervention strategies.

## Limitations

This study has several limitations that should be acknowledged. First, key clinical variables such as age of onset, mode of onset, and illness course were obtained through retrospective self-reports, which may be subject to recall bias and affect the accuracy of the data. Second, 51 patients were excluded from the final sample due to their inability to complete the diagnostic interview and assessment procedures, which, although necessary for ensuring data reliability, may have had a minor impact on the representativeness of the sample. Third, although the sample size was adequate for preliminary analysis, it may not be sufficient to generalize the findings to broader clinical populations. Fourth, cultural factors may have influenced the diagnosis of OCPD, particularly with regard to perfectionism and control traits that may be shaped by Turkish societal norms. Fifth, the study focused only on the presence or absence of OCPD and did not evaluate other personality disorders (e.g., avoidant, dependent), which may also contribute to symptom presentation and clinical outcomes. Lastly, diagnoses were based solely on DSM-5 criteria and structured clinical interviews; no additional confirmatory methods such as informant reports or longitudinal follow-up were used. Future studies with larger, more diverse samples and longitudinal designs are needed to confirm and expand upon these findings.

## Data Availability

The raw data supporting the conclusions of this article will be made available by the authors, without undue reservation.
